# Role of lysosomal enzymes released by alveolar macrophages in the pathogenesis of the acute phase of hypersensitivity pneumonitis

**DOI:** 10.1155/S0962935195000081

**Published:** 1995-01

**Authors:** J. L. Pérez-Arellano, M. N. Barrios, T. Martín, M. L. Sánchez, J. M. González Buitrago, A. Jiménez

**Affiliations:** 1 Departamento de Medicina Facultad de Medicina Universidad de Salamanca Avda Campo Charro s/n Salamanca 37007 Spain; 2 Servicio de Bioquimica Hospital Virgen de la Vega Paseo de San Vincente s/n Salamanca 37000 Spain

## Abstract

Hydrolytic enzymes are the major constituents of alveolar macrophages (AM) and have been shown to be involved in many aspects of the inflammatory pulmonary response. The aim of this study was to evaluate the role of lysosomal enzymes in the acute phase of hypersensitivity pneumonitis (HPs). An experimental study on AM lysosomal enzymes of an HP-guinea-pig model was performed. The results obtained both *in vivo* and *in vitro* suggest that intracellular enzymatic activity decrease is, at least partly, due to release of lysosomal enzymes into the medium. A positive but slight correlation was found between extracellular lysosomal activity and four parameters of lung lesion (lung index, bronchoalveolar fluid total (BALF) protein concentration, BALF LDH and BALF alkaline phosphatase activities). All the above findings suggest that the AM release of lysosomal enzymes during HP is a factor involved, although possibly not the only one, in the pulmonary lesions appearing in this disease.


Mediators of Inflammation 4, 43–48 (1995)

### Role of lysosomal enzymes released by alveolar macrophages in the pathogenesis of the acute phase of hypersensitivity pneumonitis

J. L. Pérez-Arellano,[1,CA] M. N. Barrios,[1] T. Martín,[1] M. L. Sánchez,[1] J. M. González Buitrago[2] and A. Jiménez[1]

[1]Departamento de Medicina, Facultad de Medicina, Universidad de Salamanca, Avda Campo Charro s/n, 37007, Salamanca, Spain. [2]Servicio de Bioquimica, Hospital Virgen de la Vega, Paseo de San Vincente s/n, 37000, Salamanca, Spain

[CA] Corresponding Author

**Hydrolytic enzymes are the major constituents of alveolar macrophages (AM) and have been shown to be involved in many aspects of the inflammatory pulmonary response. The aim of this study was to evaluate the role of lysosomal enzymes in the acute phase of hypersensitivity pneumonitis (HPs). An experimental study on AM lysosomal enzymes of an HP-guinea-pig model was performed. The results obtained both *in vivo* and *in vitro* suggest that intracellular enzymatic activity decrease is, at least partly, due to release of lysosomal enzymes into the medium. A positive but slight correlation was found between extracellular lysosomal activity and four parameters of lung lesion (lung index, bronchoalveolar fluid total (BALF) protein concentration, BALF LDH and BALF alkaline phosphatase activities). All the above findings suggest that the AM release of lysosomal enzymes during HP is a factor involved, although possibly not the only one, in the pulmonary lesions appearing in this disease.**

**Key words**: Alveolar macrophage, Hydrolytic enzymes, Hypersensitivity pneumonitis

### Introduction

The pulmonary interstitial–alveolar region is continuously attacked by antigens from the exterior and from the pulmonary circulation. Alveolar macrophages (AMs) are the cells found in the highest proportions in the alveolus both in healthy individuals and in patients suffering from diffuse alveolo-interstitial disease. These cells play a crucial role in the induction and resolution of inflammatory pulmonary injury. In order to participate adequately in the inflammatory response, AMs release large numbers of soluble mediators (i.e. hydrolytic enzymes, oxygen free radicals, cytokines, arachidonic acid metabolites) which can injure the lung parenchyma.

Hydrolytic enzymes are the major constituents of AMs and have been shown to be involved in many aspects of the inflammatory pulmonary response in addition to their better known role in bactericidal processes.[1] Although it is known that in most diffuse interstitial pulmonary diseases there is recruitment and activation of macrophages,[2] no systematic study has been conducted on the role of hydrolytic enzymes in these diseases.

Hypersensitivity pneumonitis (HP) is a group of lung diseases that result from repeated exposure to some antigenic organic dusts. The disease mainly affects the distal airways and is characterized by interstitial and alveolar inflammation often associated with granulomas. HP is a model of considerable interest in the study of the pathogenic mechanisms of interstitial lung diseases since in this disease alveolitis is more intense. Various HP models have been described; of special relevance to the study of macrophage activity are those developed in guinea-pigs.[3] We therefore used an experimental model of HP in order to determine the role of AM lysosomal enzymatic activity in the pathogenesis of alveolar damage.

### Materials and Methods

*HP experimental model: Faenia rectivirgula* (CECT 3223) was kindly provided by the Departamento de Microbiología (Facultad de Ciencias Biológicas, Universidad de Valencia). This strain corresponds to ATCC 15347. *F. rectivirgula* was grown in trypticase–soy broth at 52°C for 6 days in a shaking incubator, harvested by centrifugation at 450 × ***g*** and the organisms washed three times with sterile saline. Cell walls were disrupted with a Polytron homogenizer (Kinematica, Kriens/Luzern, Switzerland) and the mixture was then sonicated (Sonifier, Branson Sonic Power Company, Danford, CT) three times (20 s periods). The lysate was lyophilized and stored in sterile vials.

Male Dunkin–Hartley guinea-pigs (200–250 g, IFFA Credo, Spain) were used for all studies. They were housed in sterile rooms and were allowed food

© 1995 Rapid Communications of Oxford Ltd

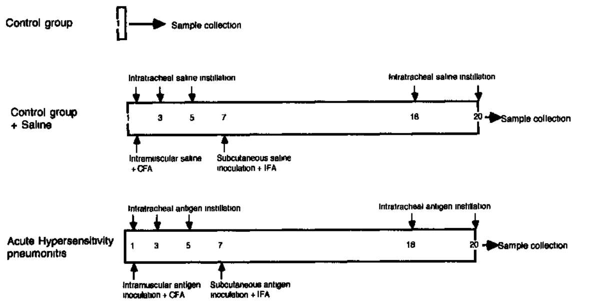

FIG. 1. Experimental protocol. CFA, complete Freund adjuvant; IFA, incomplete Freund adjuvant. The figures represent time in days.

and water *ad libitum*. Intramuscular injection of ketamine (100 mg/kg body weight) was used for sedation and intraperitoneal sodium pentobarbital (100 mg/ml body weight) for anaesthesia. HP was induced according to the protocol of Schuyler and Crooks[4] as shown in Fig. 1.

Lyophilized *F. rectivirgula* antigen was resuspended in pyrogen-free saline at 4 mg/ml and administered intratracheally at 3.6 mg/kg body weight. For intramuscular or subcutaneous inoculation the antigen (1.6 mg) was emulsified in 400 µl of complete (Sigma F-5881) or incomplete Freund adjuvant (Sigma F-5506) respectively. A group of normal guinea-pigs and a control group (intratracheal and parenteral inoculation of pyrogen-free saline) was included in the study. All procedures were performed using standard sterile materials. All animals were screened for non-apparent infections by *Paraspidodera uncinata*.[5]

*Collection of samples:* Two hours after the last intratracheal challenge with *Faenia rectivirgula* antigen, the guinea-pigs were sacrificed. Bronchoalveolar lavage was performed using 12 ml of pyrogen-free saline in three aliquots (3 × 4 ml). The fluid was filtered through sterile gauze and then centrifuged at 500 × **g** for 10 min at 4°C. The supernatant was immediately frozen at –70°C and the cells were resuspended in 2 ml of phosphate-buffered saline. Blood was obtained by direct intracardiac puncture and was used to obtain serum, which was then stored at –70°C. The lungs were then dissected, weighed, placed in 10% formaldehyde and processed for histological examination.

*Cytological studies of bronchoalveolar lavage:* BAL cells were counted on a haemocytometer and viability was assessed by Trypan blue exclusion. Cytocentrifuge preparations were stained with Diff-Quik® and a differential count was performed, 200 cells being counted by two observers. Nonspecific esterase, β-glucuronidase and tartrate-resistant acid phosphatase stains were performed using commercially available reagents (Sigma Chemical Company). Results for β-glucuronidase and tartrate-resistant acid phosphatase stains were expressed both as the percentage of positive cells and as an intensity score according to Roodman.[6]

*Studies of bronchoalveolar lavage supernatant:* BAL fluid aliquots were used for the determination of protein concentration, LDH activity, alkaline phosphatase activity and tartrate-sensitive acid phosphatase activity. All measurements were carried out on the same day and under the same conditions to avoid interassay variability.

Total protein concentrations were assayed by an automated colorimetric method[7] (Boehringer Mannheim). The detection limit was 65 µg/ml and the intraassay coefficient of variation was 3.45%.

All enzyme activities were measured at 37°C by automated colorimetric methods[8,9] using reagents from Boehringer Mannheim and results were reported as U/l. The detection limit for LDH (EC 1.1.1.27) was 0.73 U/l and the intraassay coefficient of variation was 2.4%. The detection limit for alkaline phosphatase (E.C. 3.1.3.1) was 0.58 U/l and the intraassay coefficient of variation was 2.26%. Tartrate-sensitive acid phosphatase activity was calculated by the difference between total and tartrate-resistant activity. The detection limit of total acid phosphatase (EC 3.1.3.2) and tartrate-resistant acid phosphatase were 0.01 U/l and 0.025 U/l, respectively, with an intraassay coefficient of variation of 1.5% and 7.27%.

*Serum studies:* Serum aliquots were used for the determination of total protein concentration, the serum electrophoretic protein profile, LDH activity, alkaline phosphatase activity and tartrate-sensitive acid phosphatase activity. All measurements were carried out on the same day and under the same conditions to avoid interassay variability.

Total protein concentrations were analysed by an automated colorimetric method[10] using reagents from Boehringer Mannheim. Concentrations were expressed in grams per decilitre. The detection limit was 0.126 g/dl and the intraassay coefficient of variation was 1.21%. Electrophoresis on cellulose acetate was used for the separation of serum proteins. The percentage of each fraction was measured automatically (Olympus Hyte-System 310) after staining protein bands with Ponceau red. Results were reported as the concentration of each fraction.

Serum enzyme activities were measured using the same techniques as for the broncho-alveolar lavage fluid. The detection limit of serum LDH was 1.7 U/l and the intraassay coefficient of variation was 0.53%. For serum alkaline phosphatase the detection limit was 1.38 U/l and the intraassay coefficient of variation was 1.1%. The detection limit of total serum acid phosphatase was 1.02 U/l and the intraassay coefficient of variation was 7.38%. Specific antibodies against *F. rectivirgula* were detected by double diffusion in agar[11] using a commercial filtered antigen (Mercia Diagnostics Limited, UK). Positive and negative control sera was also provided by Mercia Diagnostics.

*Histological study:* Lungs were prepared for histological examination by fixation with 10% buffered formalin (pH = 7). The tissue was sectioned by normal procedures and stained with haematoxylin and eosin.

*Lung index:* The lungs, with trachea intact, were isolated and weighed to determine lung index, defined as follows:[12]

$$\text{Lung index} = \frac{\text{Lung weight/Body weight test animal}}{\text{Lung weight/Body weight normal animal}}$$

*In vitro Secretion of acid phosphatase by guinea-pig AMs:* Alveolar macrophages (96 to 98% pure) were recovered from normal guinea-pigs by BAL which was performed using 20 ml of pyrogen-free saline in five aliquots (4 × 5 ml). The BAL fluid was first filtered through sterile gauze and centrifuged on a Ficoll-Hypaque gradient (450 × **g**, 30 min at 20°C). After separation and two washings with sterile PBS, cells were resuspended in RPMI 1640 supplemented with 10% FCS (foetal calf serum), 2 mM glutamine and penicillin–streptomycin (complete medium).

Cells were plated in four culture plates (Costar) in 1 ml of complete medium and allowed to adhere for 2 h at 37°C, 5% $CO_2$. Non-adherent cells were removed and 1 ml of fresh medium was added. Cells were incubated alone or with opsonized zymosan (400 μg/ml). After 24 h at 37°C, 5% $CO_2$, the supernatant was collected and 400 μl of sucrose–EDTA buffer added to the cell monolayer. The cells were lysed by six freeze–thawing cycles. Acid phosphatase and LDH were measured in the supernatant and in the cell lysate.

*Statistical analysis:* Data are expressed as means ± S.E. Statistical analysis was performed using a non-parametric test (Kruskal–Wallis test for global comparison, Mann–Whitney test for two-groups comparison and Spearman rank correlation test) using the Statworks™ and Statview™ software programs for an Apple Macintosh™ computer. For comparisons, $p$ values < 0.05 were adopted as significant.

### Results

*Validation of the animal model:* In order to ensure that the animal model did indeed make use of a disease characteristic of HP, various parameters were evaluated. Initially it was observed that hypergammaglobulinaemia (Table 1) and precipitating antibodies to *Faenia rectivirgula* appeared in the serum of all the guinea-pigs inoculated intratracheally with particulate antigen. Moreover, there was an intense alveolitis (mainly mononuclear phagocytes and eosinophils) and a rise in the lung index and protein concentrations in the BALF (Table 1). Finally, in the group of animals subjected to the HP experimental model, histological study of the lung revealed an intense lymphomonocytic infiltrate, with the formation of granulomas and intense alveolitis. Thus, although there are no agreed criteria in the literature for defining HP, our experimental model combines the individual criteria used separately by other authors, and we are therefore confident that it is adequate.[13–15]

**Table 1.** Validation of animal model

| | Groups | | | | | |
|---|---|---|---|---|---|---|
| | Control ($n$ = 6) | | Control + saline ($n$ = 6) | | Acute HP ($n$ = 6) | |
| | Mean | S.E.M. | Mean | S.E.M. | Mean | S.E.M. |
| Total cell counts (× $10^6$/ml) | 0.49 | 0.26 | 0.89 | 0.44 | 1.96[a,b] | 1.18 |
| BALF alveolar macrophages (× $10^4$/ml) | 49.2 | 32.0 | 77.2 | 37.8 | 97.3[a] | 37.3 |
| BALF lymphocytes (× $10^4$/ml) | 0.1 | 0.3 | 0.2 | 0.4 | 2.16[a,b] | 1.1 |
| BALF neutrophils (× $10^4$/ml) | 0.2 | 0.4 | 0.1 | 0.2 | 0.4 | 1.2 |
| BALF eosinophils (× $10^4$/ml) | 28.6 | 28.3 | 11.0 | 5.1 | 109.1[a,b] | 95.4 |
| Lung index | 0.9 | 0.12 | 1.01 | 0.08 | 1.70[a,b] | 0.30 |
| BALF total protein (μg/ml) | 80 | 15.5 | 91 | 28.5 | 191[a,b] | 133 |
| Serum γ-globulin (g/dl) | 0.17 | 0.03 | 0.17 | 0.04 | 0.37[a,b] | 0.10 |

HP, hypersensitivity pneumonitis; BALF, bronchoalveolar lavage fluid.
[a]$p < 0.05$ compared with control group. [b]$p < 0.05$ compared with control + saline group.

*AM lysosomal enzymatic activity:* Intracellular lysosomal enzymatic activities were studied by cytochemical methods because the number of cells were too low for use of biochemical methods and it is reported that cytochemical methods are more appropriate for checking the activity of isolated cell types.[16]

The proportion of β-glucuronidase positive and acid phosphatase positive cells and the score for these enzymatic activities are shown in Fig. 2. No significant differences in β-glucuronidase score, the proportion of positive β-glucuronidase and acid phosphatase cells were found between the control group and the control plus saline group, although all enzymatic activities were significantly decreased in the acute HP group compared with the other groups.

Extracellular lysosomal enzyme activity (both in BALF and serum) is summarized in Table 2. There were no significant differences in BALF total, tartrate-sensitive and tartrate-resistant acid phosphatase between the control group and the control plus saline group, although all enzymatic activities were significantly increased ($p < 0.05$) in the acute HP group compared with the other groups. No correlation was detected between intra- and extracellular lysosomal enzymatic activities and there were no differences in serum acid phosphatase.

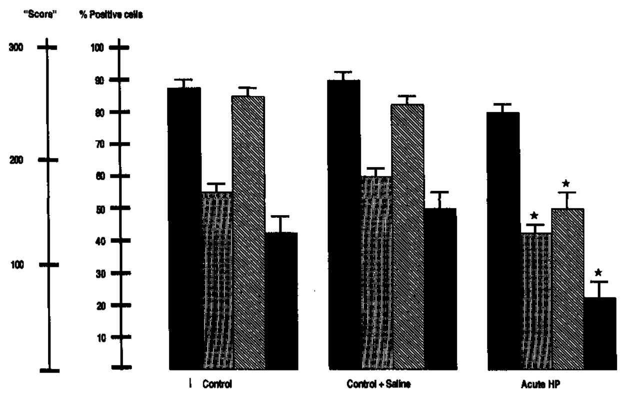

FIG. 2. Intracellular AM lysosomal enzymatic activities. Data are mean ± S.E.M. of six guinea-pigs/group and were analysed by Kruskal–Wallis test and Mann–Whitney test. Stars (★) represent significant differences ($p < 0.05$) with respect to control group and control + saline group. Symbols are: ■ β-glucuronidase (+) cells; ▒ β-glucuronidase 'score'; ▨ acid phosphatase (+) cells; and ▥ acid phosphatase 'score'. HP = hypersensitivity pneumonitis.

These findings, considered as a whole, suggest that *F. rectivirgula* induces an enzymatic lysosomal release *in vivo*. In order to confirm this interpretation, AMs from healthy guinea-pigs were incubated in the presence of a particulate antigen, since under these conditions enzymes are released into the extracellular environment.[17] Simultaneous measurement of LDH and acid phosphatase in the supernatant and in the cell lysate showed that the release of lysosomal enzymes into the culture medium was an active process since 25–45% acid phosphatase was released compared with 10–13% of LDH.

*Relationship between lysosomal enzymatic activity and lung lesion indexes:* Having demonstrated that there was an increase in extracellular lysosomal enzymatic activity in the NH group, we next studied the relationship between this activity and various parameters of pulmonary parenchymal lesion (lung index, BALF protein, LDH and alkaline phosphatase in the BAL). A slight, but significant, positive correlation was found between tartrate-sensitive acid phosphatase and lung index ($Rs = 0.40$, $p = 0.042$) and BALF protein concentration ($Rs = 0.41$, $p = 0.039$).

LDH and alkaline phosphatase activities in BAL were both significantly increased in the HP group compared with the other groups (Fig. 3). No significant differences were found in serum LDH levels (data not shown) and alkaline phosphatase values were significantly decreased in the HP group (mean = 231 U/l; S.E.M. = 56) compared with the control (mean = 618 U/l; S.E.M. = 168) and control plus saline (mean = 469; S.E.M. = 61) groups. A slight, but significant, positive correlation was found between tartrate-sensitive acid phosphatase and BALF LDH

**Table 2.** Extracellular lysosomal enzymatic activity

| Acid phosphatase | Groups | | | | | |
|---|---|---|---|---|---|---|
| | Control ($n = 6$) | | Control + saline ($n = 6$) | | Acute HP ($n = 6$) | |
| | Mean | S.E.M. | Mean | S.E.M. | Mean | S.E.M. |
| Total BALF acid phosphatase (U/l) | 1.27 | 0.26 | 1.46 | 0.16 | 2.92[a,b] | 0.66 |
| Tartrate resistant BALF acid phosphatase (U/l) | 0.45 | 0.07 | 0.42 | 0.01 | 0.76[a,b] | 0.11 |
| Tartrate sensitive BALF acid phosphatase (U/l) | 0.82 | 0.21 | 1.04 | 0.15 | 2.28[a,b] | 0.78 |
| Total serum acid phosphatase (U/l) | 31.8 | 3.05 | 19.22 | 1.21 | 24.29 | 2.07 |

HP, hypersensitivity pneumonitis; BALF, bronchoalveolar lavage fluid.
[a]$p < 0.05$ compared with control group. [b]$p < 0.05$ compared with control + saline group.

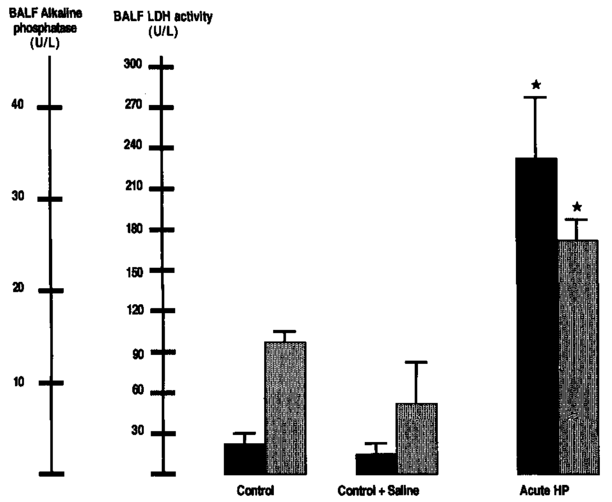

FIG. 3. BALF LDH and alkaline phosphatase enzymatic activities. Data are mean ± S.E.M. of six guinea-pigs/group and were analysed by Kruskal–Wallis test and Mann–Whitney test. Stars (★) represent significant differences ($p < 0.05$) with respect to control group and control + saline groups. No significant differences were found between control and control + saline groups. Symbols are: ■ LDH activity; ▒ alkaline phosphatase activity; BALF = bronchoalveolar lavage fluid; HP = hypersensitivity pneumonitis.

activity ($Rs = 0.42$, $p = 0.036$) and between tartrate-sensitive acid phosphatase and BALF alkaline phosphatase ($Rs = 0.45$, $p = 0.021$).

### Discussion

Accumulation of inflammatory cells in the alveolar region of the lung is a key event in pathogenesis of diffuse pulmonary interstitial disease. In HP the AMs are activated, and in response to the causal antigens they release a large number of inflammatory mediators (i.e. lysosomal enzymes, oxygen free radicals, cytokines, arachidonic acid metabolites) capable of injuring the lung parenchyma. Lysosomal acid hydrolases form a group of enzymes whose function is to degrade the material ingested in the phagocytic vacuole. However, lysosomal enzymes can sometimes gain access to the extracellular medium where they exert a lytic action by degrading collagen and proteoglycans under acid pH conditions (as happens at the focus of inflammation).[18] Lysosomal intracellular activity depends on the equilibrium between their production and the release into the extracellular medium. In turn, production depends on cellular differentiation and the degree of activation; levels of lysosomal hydrolases vary in different subpopulations of AMs (minimum activity corresponding to the most immature forms)[19] while enzymatic activity increases upon activation by various physical, chemical and biological agents.[20–22] The other factor affecting intracellular acid hydrolase activity is the release of enzymes into the extracellular medium mediated by particulate[16,23–25] or immunological stimuli.[26,27]

Our results confirm the data obtained in humans, there being a decrease in the intracellular enzymatic activity of the guinea-pig AMs.[3] The results obtained both *in vivo* and *in vitro* suggest that this decrease is, at least partly, due to release of lysosomal enzymes into the medium. However, the lack of correlation between the intra- and extracellular activities indicates that other mechanisms may affect the decrease in intracellular enzymatic activity. In this sense, the very low levels of acid phosphatase compared with β-glucuronidase activity afford complementary data. In general, it is assumed that although the release of lysosomal enzymes takes place as a whole, there are substantial differences when several activities in a single process are measured simultaneously,[28] perhaps depending on the different species-specific enzymatic content or the presence of specific inhibitors.[29] In the case of AMs, another factor influencing these differences is the presence of specific acid phosphatase isoenzymes with special characteristics. In particular, these cells possess an isoenzyme with high electrophoretic mobility, which is tartrate-resistant (band 5).[30] The presence of tartrate-resistant acid phosphatase in cells of the MPs has been interpreted as a marker of differentiation[30] and indeed it has been shown that this activity is decreased in active sarcoidosis, a disease in which there is considerable monocyte recruitment to the lung.[31] Accordingly, the decrease in intracellular acid phosphatase activity involves not only release into the extracellular environment but also an increase in the recruitment of young MPs' cells with lower enzymatic activity.

The last aspect considered in this work was the relationship between extracellular lysosomal enzymatic activity and different parameters of lung lesion (lung index, total BALF protein concentration, LDH activity and alkaline phosphatase activity). Lung index[12,32] and BALF total protein concentration are two useful but nonspecific parameters for evaluating lung lesions in interstitial diseases. The acute HP group showed elevated values of lung index and BALF total protein concentrations, together with a positive although low correlation with extracellular lysosomal activity. BALF lactic dehydrogenase activity measurement has been used as a parenchymal lung lesion parameter in diverse experimental animal models[33] and in human diseases.[34] In our study maximum BALF LDH values were detected in the acute HP group. We can reasonably reject that LDH come from plasma since the plasma LDH values were similar in all the experimental groups. Alkaline phosphatase activity in the lower respiratory tract is limited to type II pneumocytes[35] and levels of this enzyme in BALF has been used as an index of pneumocyte lesion and/or proliferation.[29,36] Since in acute HP there is a hypertrophy of type II pneumocytes as well as marked structural alterations in these cells,[37] we used this measurement to evaluate these events. Our results revealed a marked rise in BALF alkaline phosphatase activity in acute HP with respect to both control groups. This increase cannot be attributed to increased serum activity since in the acute HP group the serum values of this enzyme actually decreased. This could be explained by the increased synthesis of macrophage cytokines[38] and/ or a fall in parathormone activity secondary to the formation of granulomas.[39] We are currently studying possible mechanisms responsible for this decrease. A positive but slight correlation was found between BALF LDH and alkaline phosphatase activities and extracellular lysosomal activity. All the above findings suggest that the release of lysosomal enzymes during HP is a factor involved, although possibly not the only one, in the pulmonary lesions appearing in this disease.

### References

1. Goldstein E. Hydrolytic enzymes of alveolar macrophage. *Rev Infect Dis* 1983; **5**: 1078–1092.
2. Fantone JC, Ward PA. Mechanisms of lung parenchymal injury. *Am Rev Respir Dis* 1984; **130**: 484–491.
3. Pérez-Arellano JL, Barrios González N, Martín Dominguez T, Sánchez Benitez de Soto ML, Jiménez Lópéz A. Experimental models of hypersensitivity pneumonitis revisited. *J Invest Allergol Clin Immunol* 1992; **2**: 219–228.
4. Schuyler M, Crooks L. Experimental hypersensitivity pneumonitis in guinea pigs. *Am Rev Respir Dis* 1989; **139**: 996–1002.
5. Conder GA, Richards IM, Jen LW, Marbury KS, Oostveen JA. Bronchoalveolar eosinophilia in guinea pigs harboring inapparent infections of *Paraspidodera uncinata*. *J Parasitol* 1989; **75**: 144–146.
6. Roodman GD, Ibbotson KJ, MacDonald BR, Kuehl TJ, Mundy GR. 1,25-Dihydroxyvitamin D3 causes formation of multinucleated cells with several osteoclast characteristics in cultures of primate marrow. *Proc Natl Acad Sci USA* 1985; **82**: 8213–8217.
7. Watanabe N, Kamei S, Okhubo A, Yamanaka M, Ohsawa S, Makino K, Tokuda K. Urinary protein as measured with pyrogallol red–molybdate complex, manually and in a Hitachi 726 automated analyzer. *Clin Chem* 1986; **32**: 1551–1554.
8. Hillmann G. Fortlaufende photometrische messung der sauren prostataphosphatase ak-tivität. *Z Klin Chem Klin Biochem* 1971; **9**: 273–274.
9. Fishman WH, Springer BJ, Brunetti R. Application of an improved glucuronidase assay method to study of human blood beta glucuronidase. *J Biol Chem* 1948; **173**: 449–456.
10. Gornall AG, Bardawill CJ, David MM. Determination of serum proteins by means of the Biuret reaction. *J Biol Chem* 1949; **177**: 751–766.
11. Johnstone A, Thorpe R. Precipitation techniques in agar and agarose. In: Johnstone A, Thorpe R, eds. *Immunochemistry in Practice*. Oxford: Blackwell Scientific Publications, 1990; 131.
12. Wilson BD, Sternick JL, Yoshizawa Y, Katzenstein AL, Moore V. Experimental murine hypersensitivity pneumonitis: multigenic control and influence by genes within the I-B subregion of the H-2 complex. *J Immunol* 1982; **129**: 2160–2163.
13. Cormier Y, Gagnon L, Berubé-Genest F, Fournier M. Sequential bronchoalveolar lavage in experimental extrinsic allergic alveolitis. The influence of cigarette smoking. *Am Rev Respir Dis* 1988; **137**: 1104–1109.
14. Kawai T, Salvaggio J, Lake W, Harris JO. Experimental production of hypersensitivity pneumonitis with bagasse and thermophilic actinomycete antigen. *J Allergy Clin Immunol* 1972; **50**: 276–288.
15. Salvaggio J, Phanuphak P, Stanford R, Bice D, Claman H. Experimental production of granulomatous pneumonitis: comparison of immunological and morphological sequeae with particulate and soluble antigens administered via the respiratory route. *J Allergy Clin Immunol* 1975; **56**: 364–380.
16. Chandrasekhar S, Mukherjee MK. Intracellular tubercle bacilli–alveolar macrophage lysosomal enzymes interaction in experimental tuberculosis. *Clin Immunol Immunopathol* 1990; **56**: 185–201.
17. Speziale SC, Smith RJ. Effects of soluble stimuli on human monocyte secretion. *Clin Immunol Immunopathol* 1985; **36**: 60–69.
18. Sibille Y, Reynolds HY. Macrophages and polymorphonuclear neutrophils in lung defense and injury. *Am Rev Respir Dis* 1990; **141**: 471–501.
19. Spiteri MA, Poulter LW. Characterization of immune inducer and suppressor macrophages from the normal human lung. *Clin Exp Immunol* 1991; **83**: 157–162.
20. Loose LD, Megirian R, Turinsky J. Biochemical and functional alterations in macrophages after thermal injury. *Infect Immun* 1984; **44**: 554–558.
21. Dogra S, Kaw JL. Changes in some histochemically demonstrable enzymes in macrophages exposed to quartz dust *in vitro*. *J Appl Toxicol* 1988; **8**: 23–27.
22. Briend Sutren MM, Le-Mano S, Rommain M, Lambre CR. Glycosidase activities in alveolar macrophages from guinea pigs stimulated with a glycoproteic complex extracted from *Klebsiella pneumoniae*. *Agents Actions* 1990; **31**: 308–312.
23. Gupta N, Garg UC, Dhand R, Kaur A, Ganguly NK. Enzyme levels in bronchoalveolar lavage fluid and serum of active pulmonary tuberculosis patients. *Enzyme* 1989; **41**: 108–111.
24. Forget G, Lacroix MJ, Calvert R, Sirois P. Measurement of beta-glucuronidase in effluent of perfused alveolar macrophages challenged with chemically modified chrysolite asbestos. *Inflammation* 1984; **8**: 123–141.
25. Paterson NAM, McIver DJL, Schurch S. Zymosan enhances leukotriene D4 metabolism by porcine alveolar macrophages. *Immunology* 1985; **56**: 153–159.
26. Thorel T, Joseph M, Tsicopoulos A, Tonnel AB, Capron A. Inhibition by neochromyl sodium of IgE-mediated activation of human mononuclear phagocytes and platelets in allergy. *Int Arch Allergy Appl Immunol* 1988; **85**: 232–237.
27. McCarthy K, Henson PM. Induction of lysosomal enzyme secretion by alveolar macrophages in response to the purified complement fragments C5a and C5a des-arg. *J Immunol* 1979; **123**: 2511–2517.
28. Kumar P, Chandrasekar S. Extracellular enzymes of pulmonary fluid and their bactericidal effects on *Mycobacterium tuberculosis*. *Acta Microbiol Hung* 1990; **37**: 39–43.
29. Henderson RF, Mauderly JL, Pickrell JA, Hahn FF, Muhle H, Rebar AH. Comparative study of bronchoalveolar lavage fluid: effects of species, age and method of lavage. *Exp Lung Res* 1987; **13**: 329–342.
30. Radzun HJ, Kreipe H, Parwaresch MR. Tartrate resistant acid phosphatase as a differentiation marker for the human mononuclear phagocyte system. *Hematol Oncol* 1983; **1**: 321–327.
31. Barth J, Kreipe H, Kiemle-Kallee J, Radzun HJ, Parwaresch MR, Petermann W. Diminished activity of tartrate resistant acid phosphatase in alveolar macrophages from patients with active sarcoidosis. *Thorax* 1988; **43**: 901–904.
32. Takizawa H, Suko M, Kobayashi N, Shoji S, Ohta K, Nogami M, *et al.* Experimental hypersensitivity pneumonitis in the mouse: histologic and immunologic features and their modulation with cyclosporin A. *J Allergy Clin Immunol* 1988; **81**: 391–400.
33. Forkert PG, Custer EM, Alpert AJ, Ansari GAS, Reynolds ES. Lactate dehydrogenase activity in mouse lung following 1,1,-dichloroethyline: index of airways injury. *Exp Lung Res* 1982; **4**: 67–77.
34. Hoffman RM, Rogers RM. Serum and lavate lactage dehydrogenase isoenzymes in pulmonary alveolar proteinosis. *Am Rev Respir Dis* 1991; **143**: 42–46.
35. Edelson JD, Shannon JM, Mason RJ. Alkaline phosphatase: a marker of alveolar type II cell differentiation. *Am Rev Respir Dis* 1988; **138**: 1268–1275.
36. Reasor MJ, Nadeau D, Hook GER. Extracellular alkaline phosphatase in the airways of the rabbit lung. *Lung* 1987; **155**: 321–335.
37. Quezada AL, Gimpel SF, Miranda DO, Las Heras JB, Andreis MC. Ultrastructural features of alveolar cells in experimental hypersensitivity pneumonitis. *Respiration* 1987; **51**: 127–136.
38. Hambleton P, Bailey NE, Fitzgeorge RB, Baskerville A. Clinical chemical responses to experimental airborne legionellosis in the guinea pig. *Br J Exp Pathol* 1985; **66**: 173–183.
39. Alberts C, Van der Bergh H. Calcium metabolism in sarcoidosis. A follow-up study with respect to parathyroid hormone and vitamin D metabolites. *Eur J Respir Dis* 1986; **68**: 186–194.

ACKNOWLEDGEMENT. The authors gratefully thank J. H. Brock (Dept Immunology, Western Infirmary, Glasgow) for the review of the manuscript and A. Muro (Departmento de Parasitología, Universidad de Salamanca) for the parasitologic evaluation of guinea-pigs.

**Received 2 August 1994; accepted in revised form 10 October 1994**
